# The Label-Free Detection and Identification of SARS-CoV-2 Using Surface-Enhanced Raman Spectroscopy and Principal Component Analysis

**DOI:** 10.3390/bios13121014

**Published:** 2023-12-05

**Authors:** Lu Zhou, Ambra Vestri, Valentina Marchesano, Massimo Rippa, Domenico Sagnelli, Gerardo Picazio, Giovanna Fusco, Jiaguang Han, Jun Zhou, Lucia Petti

**Affiliations:** 1 Institute of Applied Sciences and Intelligent Systems of CNR, 80072 Pozzuoli, Italy; zhoulu@tju.edu.cn (L.Z.); ambra.vestri@isasi.cnr.it (A.V.); valentina.marchesano@isasi.cnr.it (V.M.); m.rippa@isasi.cnr.it (M.R.); domenico.sagnelli@isasi.cnr.it (D.S.); 2 Center for Terahertz Waves and College of Precision Instrument and Optoelectronics Engineering, Tianjin University, Tianjin 300072, China; jiaghan@tju.edu.cn; 3 Istituto Zooprofilattico Sperimentale del Mezzogiorno, 80055 Portici, Italy; gerardo.picazio@izsmportici.it (G.P.); giovanna.fusco@izsmportici.it (G.F.); 4 Department of Microelectronic Science and Engineering, School of Physical Science and Technology, Ningbo University, Ningbo 315211, China

**Keywords:** surface-enhanced Raman scattering, SARS-CoV-2, principal component analysis

## Abstract

The World Health Organization (WHO) declared in a May 2023 announcement that the COVID-19 illness is no longer categorized as a Public Health Emergency of International Concern (PHEIC); nevertheless, it is still considered an actual threat to world health, social welfare and economic stability. Consequently, the development of a convenient, reliable and affordable approach for detecting and identifying SARS-CoV-2 and its emerging new variants is crucial. The fingerprint and signal amplification characteristics of surface-enhanced Raman spectroscopy (SERS) could serve as an assay scheme for SARS-CoV-2. Here, we report a machine learning-based label-free SERS technique for the rapid and accurate detection and identification of SARS-CoV-2. The SERS spectra collected from samples of four types of coronaviruses on gold nanoparticles film, fabricated using a Langmuir–Blodgett self-assembly, can provide more spectroscopic signatures of the viruses and exhibit low limits of detection (<100 TCID_50_/mL or even <10 TCID_50_/mL). Furthermore, the key Raman bands of the SERS spectra were systematically captured by principal component analysis (PCA), which effectively distinguished SARS-CoV-2 and its variant from other coronaviruses. These results demonstrate that the combined use of SERS technology and PCA analysis has great potential for the rapid analysis and discrimination of multiple viruses and even newly emerging viruses without the need for a virus-specific probe.

## 1. Introduction

Over the past few years, the Severe Acute Respiratory Syndrome Coronavirus 2 (SARS-CoV-2) responsible for the Coronavirus Disease 2019 (COVID-19) has reshaped our way of life, resulting in substantial health, social and economic problems [1,2,3]. On 30 January 2020, COVID-19 was designated as a Public Health Emergency of International Concern (PHEIC) by the World Health Organization (WHO) and categorized as a pandemic after nearly a month (11 March 2020) [4,5]. Only on 5 May 2023, after a span of over 3 years, the infamous disease ceased to be considered a PHEIC, as announced by the WHO Director-General, but it persists as a current and impactful health issue [6]. This extremely challenging outbreak has taught us the critical importance of proactive measures to prevent and manage public health emergencies, including the necessity of prompt and effective deployable testing for the early detection of emerging infectious diseases [7]. Usually, the clinical symptoms of COVID-19 closely resemble those of other respiratory viruses. Therefore, rapid, accurate and cost-effective diagnosis and identification is essential for monitoring SARS-CoV-2 and its variants. To date, the established standard procedure for the early-stage detection of COVID-19 is based on Reverse-Transcription Polymerase Chain Reaction (RT-PCR) [8]. Other relevant techniques to aid in monitoring the illness are available, such as the following: chest Computed Tomography (CT), Lateral Flow Assay (LFA), Enzyme-Linked Immunosorbent Assay (ELISA), Reverse Transcription Loop-mediated Isothermal Amplification (RT-LAMP), Clustered Regularly Interspaced Short Palindromic Repeats (CRISPR), etc. [9,10,11,12,13]. These different methods give different detection parameters, such as detection specificity, accuracy, sensitivity, cost, throughput and so on; additionally, most of them are expensive and time consuming and require specialist equipment and/or trained staff. Therefore, it is essential to develop alternative tests which are faster, are simpler and have the potential to identify multiple viruses.

To overcome certain limitations of conventional techniques, different types of biosensors have been developed [14,15,16,17,18]. Among them, label-free surface-enhanced Raman scattering (SERS)-based detection has been accepted as a promising tool, owing to its ability to provide ultra-sensitive vibration fingerprint information in a noninvasive way [18,19]. Typically, the enhancement of Raman signals is ascribed to two primary mechanisms: electromagnetic (EM) enhancement, which is the dominant mechanism, and chemical enhancement [20,21]. The main mechanism is based on the excitation of Localized Surface Plasmon Resonance (LSPR) in nanostructured metal, leading to the generation of “hotspot” regions, where the electric field in the vicinity of the nanostructure surface is significantly intensified [22,23]. When the analyte enters a hotspot, its Raman signal can undergo remarkable enhancement, reaching magnitudes of millions and yielding a plethora of chemically specific signals crucial for species identification. Moreover, without any sample pretreatment, the SERS fingerprint information of an analyte can be directly captured to achieve rapid and real-time detection. Therefore, the design and fabrication of a simple, highly sensitive, reproducible and stable SERS substrate is indispensable in the potential practical application of SERS. Additionally, an important point to consider is that, even though a Raman signal can be amplified millions of times, the similar chemical composition and small Raman scattering cross-sections of viruses may interfere in the identification of multiple viruses [24]. Thus, further analysis using machine learning, such as principal component analysis, can improve the sensitivity, selectivity and specificity of the detection approach to overcome the limitations mentioned above.

Herein, we developed an assay platform for the rapid detection and identification of SARS-CoV-2 and its variants through combining SERS and Principal Component Analysis (PCA). We fabricated a highly effective, easy, pure SERS substrate through the liquid/liquid interfacial self-assembly of gold nanoparticles (Au NPs) to amplify the faint Raman signal of viruses and minimize interference from the substrate background. Achieving an experimental limit of detection of 100 TCID_50_/mL (50% tissue culture infectious dose), we were able to directly read the characteristic signal of four types of viruses (SARS-CoV-2, human coronavirus OC43, SARS-CoV-2 Omicron variant BA. 5.1 and pantropic canine coronavirus) from the SERS spectra. Subsequently, PCA was utilized to extract key spectroscopic information from the minor differences in SERS spectra, allowing for the identification of four different types of coronaviruses. Our results show that combining SERS with PCA could serve as an efficient testing platform to quickly detect and identify common viruses, even newly emerging ones, without the need for probes or labels.

## 2. Materials and Methods

### 2.1. Chemicals

Hydrogen tetrachloroaurate (III) trihydrate (HAuCl_4_·3H_2_O), trisodium citrate (Na_3_C_6_H_5_O_7_·2H_2_O, ≥99%) and toluene were acquired from Carlo Erba Reagents, while 4-mercaptobenzoic acid (4MBA, 99%) was from Sigma-Aldrich. Sulfuric acid (H_2_SO_4_, 90–98%) was purchased from Alfa Aesar, and hydrogen peroxide (H_2_O_2_, 30%) was obtained from VWR Chemicals. SARS-CoV-2 (VR-1986HKTM) and human coronavirus OC43 (HoV-OC43, VR-1558TM) were obtained from ATCC. The isolation of SARS-CoV-2 variant Omicron BA. 5.1 was carried out from a nasopharyngeal sample conferred at the Istituto Zooprofilattico Sperimentale del Mezzogiorno (Portici, Italy) from the COVID-19 hospital “M. Scarlato” (Scafati, Italy). Pantropic canine coronavirus (pantropic CCoV) isolation was performed from a 2014 sample derived from a client-owned Pomeranian dog previously examined by Alfano et al., 2020 [25]. Additional details about isolations of SARS-CoV-2 variant Omicron BA. 5.1 and pantropic CCoV can be found in Supplementary Information Section S1.

### 2.2. Treatment of the Silicon Wafer

A pristine silicon wafer was treated in a solution of H_2_SO_4_:H_2_O_2_ (7:3 by volume) maintained at room temperature for 2 h, employing a conventional hydrophilic treatment. This was followed by a thorough rinse in deionized water and nitrogen gas drying.

### 2.3. Synthesis of Gold Nanoparticles (Au NPs)

Citrate-stabilized Au nanoparticles were synthesized by the reduction of tetrachloroauric acid [26,27]. A wine-red solution was achieved by adding 1 mL of 1% sodium citrate to 100 mL of a 10^−4^ g/mL HAuCl_4_ boiling solution while stirring and boiling the mixture for 10 min. Upon boiling, 1 mL of sodium citrate solution (1%) and 1 mL of HAuCl_4_ solution (10^−4^ g/mL) were then added to the mixture every 2 min, and this step was repeated 3 times. The mixture was heated at 100 °C for an additional 20 min and then cooled naturally. After the end of the reaction, 24 mL of Au NPs were centrifuged at 8000 rpm for 10 min to collect precipitates and then redispersed into 4 mL of ethanol for further use.

### 2.4. SERS Substrate Fabrication

The SERS substrate fabrication was realized through the self-assembly of Au NPs at the liquid/liquid interface, exploiting a modified procedure [28]. Initially, 8 mL of toluene was gently added over the surface of 80 mL of water to establish an immiscible toluene/water liquid interface. Then, 4 mL of Au NPs in ethanol was carefully added to this interface using a mechanical syringe pump (flow rate of 3 mL/h). As the toluene evaporated, the Au NPs spontaneously self-assembled, forming a monolayer film at the toluene/water interface. At the end, the monolayer was moved onto silicon wafer surface.

### 2.5. SERS Measurement

SERS spectra were recorded using a Raman spectrometer (QE Pro-Raman, Ocean Optics, Dunedin, FL, USA) coupled with an Olympus BX51 microscope and a semiconductor laser emitting at a wavelength of 785 nm. The spectrometer was also equipped with a grating featuring 1200 lines/mm and a 50 μm input slit. An acquisition time of 10 s, a 50× objective lens (N.A. = 0.75) and a laser power set within the range of 10 mW were also employed.

### 2.6. SERS Detection of Virus

As a standard practice, 5 µL of a virus sample was dropped and dried on the Au film at room temperature (about 30 min). This produced a coffee-ring during drying. The regular coffee-ring effect can enrich analyte at the edge of the coffee-ring. After that, we collected 20 SERS spectral data of viruses from 20 different points in the coffee-ring area and an average of multiple tests. The desired titer of virus particles was added to water and used as a solvent, and the concentration was diluted up to 10 PFU/mL.

## 3. Results and Discussion

### 3.1. Substrate Preparation and Characterization

**Figure 1:**
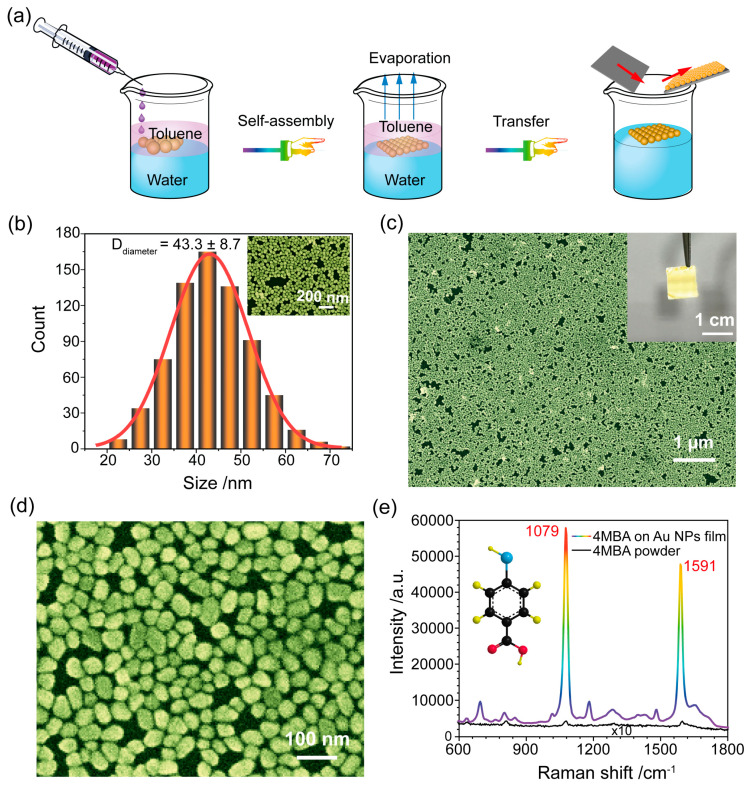
(**a**) A schematic illustration of the SERS substrate fabrication by Au NP self-assembly at the liquid/liquid interface. (**b**) The size distribution of the prepared Au NPs. The inset is the SEM image of the Au NPs. (**c**,**d**) SEM images of self-assembled Au NPs film at different magnifications. The inset is the picture of self-assembled Au NPs film on silicon wafer. (**e**) SERS spectrum of a 4MBA monolayer adsorbed on Au NPs film substrate and the normal Raman spectrum of 4MBA powder.

Monodisperse Au NPs were synthesized via the sodium citrate reduction method, and, subsequently, a large area of the Au NPs monolayer film was obtained via a liquid/liquid interfacial self-assembly strategy, which is based on the Marangoni effect [29] (see Figure 1a). Figure 1b provides the size distribution of monodisperse Au NPs by using Nano Measurer 1.2.5, according to the SEM images (Figure 1b). The mean size of the prepared Au NPs was 43.3 ± 8.7 nm. A large-area assembly of Au NPs film transferred onto Si wafer could be achieved with an area of 1 × 1 cm^2^, as shown in Figure 1c. Moreover, the Au NPs monolayer films were characterized by SEM images, as shown in Figure 1c,d. It can be easily found that the Au NPs films were well self-assembled, without obvious aggregates and large voids. As a result, these were prerequisites for good uniformity and reproducibility as SERS substrates in this study.

In addition, the SERS performance of the self-assembly Au NPs film was investigated using the 4MBA molecule as a Raman label. In Figure 1e, the SERS spectra of 4MBA exhibits distinctive fingerprint features. A prominent peak at 1079 cm^−1^ associated with in-plane ring breathing and ν(C-S) modes and a Raman peak at 1591 cm^−1^ linked to the aromatic ring ν(C-C) vibrational mode are present [30,31]. We used the SERS and normal Raman spectra of 4MBA (Figure 1e) to calculate the Enhancement Factor (EF) of the SERS substrate according to our prior works [32,33] (see Supplementary Information Section S2 for detailed information). The EF value was determined to be 3.3 × 10^7^, indicating that the prepared SERS substrate was characterized by high sensitivity.

**Figure 2:**
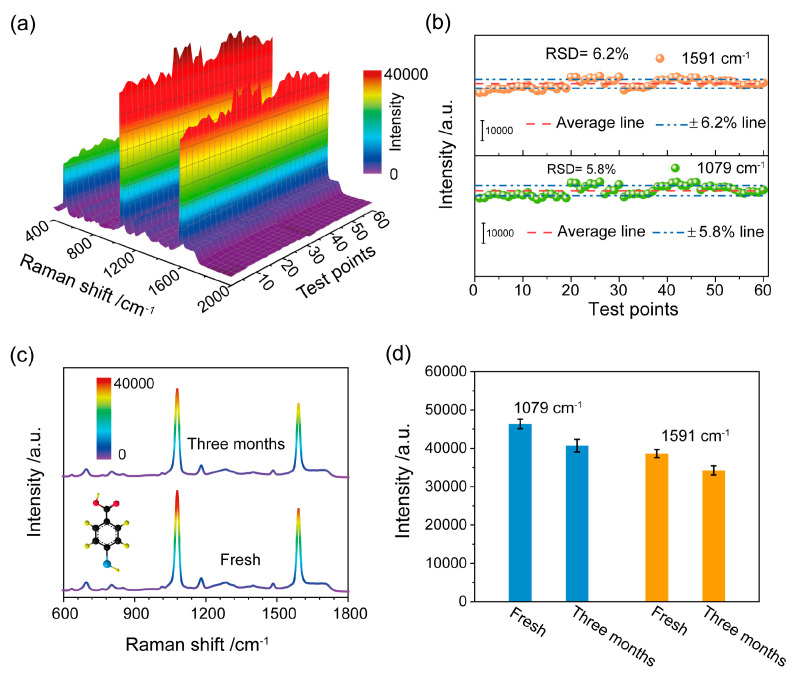
(**a**) SERS spectra of 4MBA absorbed on Au NPs film substrate at 60 random test points on the substrate; (**b**) SERS intensities of the peaks at 1079 cm^−1^ and 1591 cm^−1^ recorded at 60 different sites on the substrate. The corresponding RSD values were also reported. (**c**) SERS spectra of 4MBA molecules on the fresh SERS substrate and after a three-month storage period and (**d**) the corresponding histogram of the intensity of two typical Raman peaks.

Apart from sensitivity, the reproducibility and stability of the SERS substrate are essential factors for practical applications. Specifically, 10 µL of 4MBA solution (10 mM) was dropped on the SERS substrate; the SERS spectra at 60 random test points on the substrate were collected and shown in Figure 2a. It could be found that the 60 random Raman spectra were all well matched, and the characteristic peak did not shift or change. Generally, the reproducibility of the SERS substrate was assessed by calculating the Relative Standard Deviation (RSD) values of Raman intensities. In this regard, the RSD values of 1079 cm^−1^ and 1591 cm^−1^ peaks were 5.8% and 6.2%, as shown in Figure 2b. Subsequently, we turned our attention to the stability of the SERS substrate. Figure 2c illustrates the SERS spectra of 4MBA, recorded on a freshly prepared Au NPs film surface and on a surface stored for three months under ambient conditions. Compared to the SERS spectrum of the fresh sample, the SERS signal of 4MBA was reduced approximately by only 10% after three months of storage (see Figure 2d). Our results clearly demonstrate that the SERS substrate had high sensitivity, fine reproducibility and stability, and it could serve as a high-performance SERS platform in practical application.

### 3.2. SERS Detection of Different Coronaviruses

**Figure 3:**
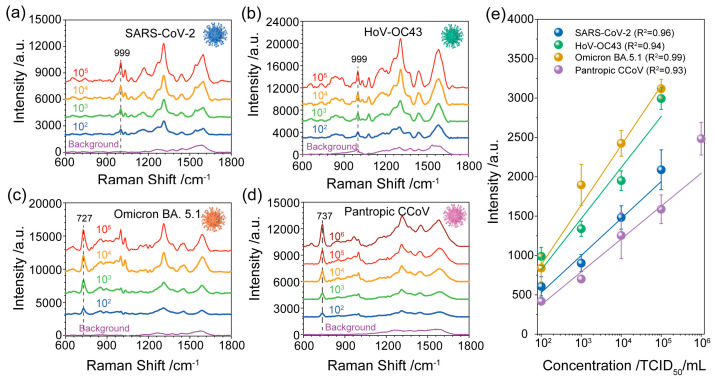
SERS spectra of (**a**) SARS-CoV-2, (**b**) HoV-OC43, (**c**) SARS-CoV-2 variant Omicron BA. 5.1 and (**d**) pantropic CCoV at different concentrations (10^2^–10^6^ TCID_50_/mL). (**e**) The corresponding standard dose–response curve of SARS-CoV-2, HoV-OC43, SARS-CoV-2 variant Omicron BA. 5.1 and pantropic CCoV is obtained by using the peak intensity at 999 cm^−1^, 999 cm^−1^, 727 cm^−1^ and 737 cm^−1^, respectively. The background is related to the blank samples without a virus.

Next, motivated by its easy preparation and high performance, we used the prepared Au NPs film substrate as a detection system for pathogenic variant identification and discrimination. We utilized four types of different viruses belonging to the Coronaviridae family: SARS-CoV-2 and the SARS-CoV-2 variant Omicron BA. 5.1—both associated with respiratory illness; human coronavirus OC43 (HoV-OC43), which is responsible for the common cold and infects humans and cattle [34]; and pantropic canine coronavirus (pantropic CCoV), which is known to infect dogs and wild carnivores. Figure 3a–d shows the averaged SERS spectra of SARS-CoV-2, HoV-OC43, SARS-CoV-2 variant Omicron BA. 5.1 and pantropic CCoV at concentrations ranging from 10^2^–10^6^ TCID_50_/mL. As we can see in Figure 3a, the intensity of SERS signals decreased with the decrease in SARS-CoV-2 concentrations. And the SERS intensity of the designated peak (999 cm^−1^), as a function of SARS-CoV-2 concentration, is displayed in Figure 3e. The corresponding standard dose–response curve was a linear relationship described by the fitting curve y = 470x − 410 (see Figure 3e), and the limit of detection (LOD) of SARS-CoV-2 was determined to be 23 TCID_50_/mL, which was calculated via the linear regression method (LOD = 3S_a_/b) [35]. Likewise, the SERS spectra and the associated dose–response curves were generated for various concentrations of HoV-OC43, Omicron BA. 5.1 and pantropic CCoV (Figure 3b–e, respectively). The LODs of HoV-OC43, Omicron BA. 5.1 and pantropic CCoV were calculated to be 69 TCID_50_/mL, 5 TCID_50_/mL and 17 TCID_50_/mL, respectively. The LODs for all four viruses were less than 100 TCID_50_/mL, proving the capability of the Au NPs film substrate to detect minimal amounts of the etiological agents under investigation.

Table 1 presents the summary of some current methods for SARS-CoV-2 detection and their LODs. It can be found that the LOD of our label-free SERS detection is equivalent to or even lower than some current common methods. However, the LOD achieved here is not so low as that of SERS label-based detection, primarily due to the smaller Raman scattering cross-section of proteins compared to Raman reporter tags [36]. Although high sensitivity was not the main goal of this work, the proposed method has a low LOD, which is more effective than other label-free SERS detection methods (see Table 1). This comparison shows that our suggested SERS-based SARS-CoV-2 detection and identification strategy has a LOD equivalent to or lower than that of common methods, and our strategy is remarkable for its rapidity and simplicity. Additionally, our LOD values estimated in the manuscript in TCID_50_/mL can be approximately converted into other units of measurement using the conversions reported in the literature (Table S1 in the Supporting Information). We report such a table in the Supporting Information (Table S1), which contains the conversions of our LOD into other unit measures.

**Figure 4:**
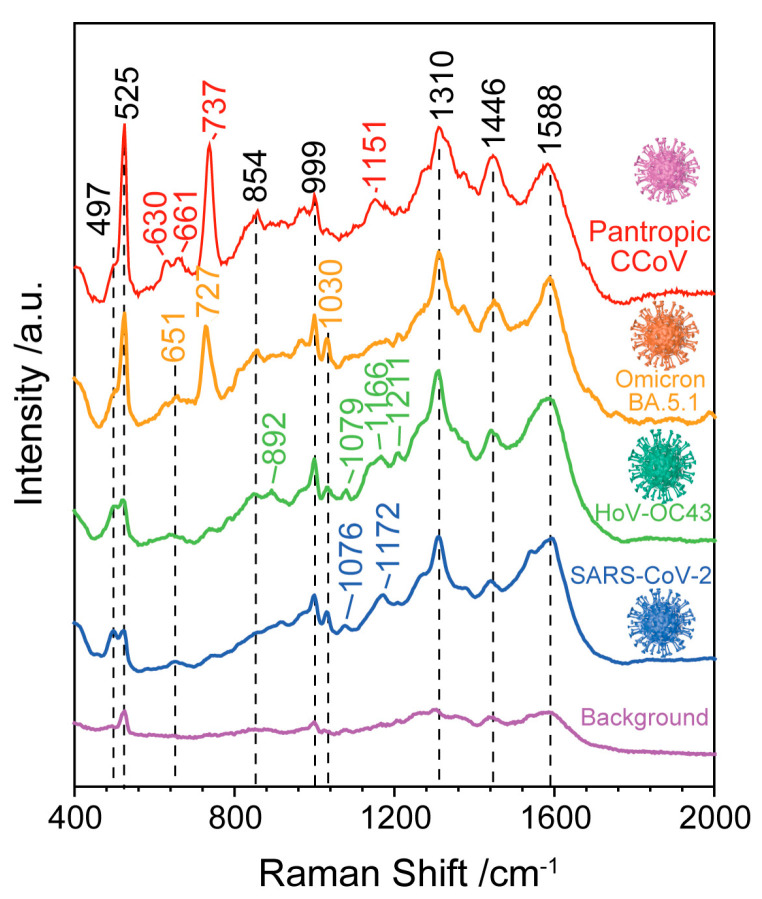
SERS spectra of SARS-CoV-2, HoV-OC43, SARS-CoV-2 variant Omicron BA. 5.1 and pantropic CCoV. The background is related to the blank samples without a virus.

Furthermore, we compared the SERS spectra collected from viral solution containing the SARS-CoV-2, HoV-OC43, SARS-CoV-2 variant Omicron BA. 5.1 and pantropic CCoV (see Figure 4). Through a comparison of the SERS spectra of the four types of viruses, the Raman modes obtained from the four viruses are slightly different. Those include the peak at 497 cm^−1^ related to the phenylalanine and tyrosine, the weak peak in the region of 651–661 cm^−1^ related to the tyrosine and cystine, two weak bands at 854 cm^−1^ and 892 cm^−1^ due to the phosphodiester stretching in DNA/RNA molecules, the band at 999 cm^−1^ related to the *δ*(C-C) aromatic ring in phenylalanine, the 1030 cm^−1^ band attribute to C=C bending mode of phenylalanine, the 1310 cm^−1^ band attributed to Amide I, the Raman mode at 1446 cm^−1^ ascribed to proteins and lipids and the Raman mode at 1588 cm^−1^ attributed to the tyrosine and tryptophan. For the viruses of SARS-CoV-2 and HoV-OC43, a unique band at 1076–1079 cm^−1^ was obtained, which is attributed to lipid C-C stretching. Compared with the SERS spectra of other viruses, sharper and higher intensity bands at 727 cm^−1^ and 737 cm^−1^ were obtained from SARS-CoV-2 variant Omicron BA. 5.1 and pantropic CCoV, which are due to the (C-S) stretching of protein. The detailed Raman modes and vibrational assignments for the four types of viruses are summarized in Table 2. Although the nucleic acid-, amide-, protein- and lipid-related components that make up the virus are visible in the SERS spectra, the minor differences between different viruses cannot be distinguished by gross visual inspection. Additionally, it is challenging to consider the entire information of the SERS spectra while conducting manual data analysis for different viruses.

### 3.3. Identification of SARS-CoV-2 Using Principal Component Analysis

**Figure 5:**
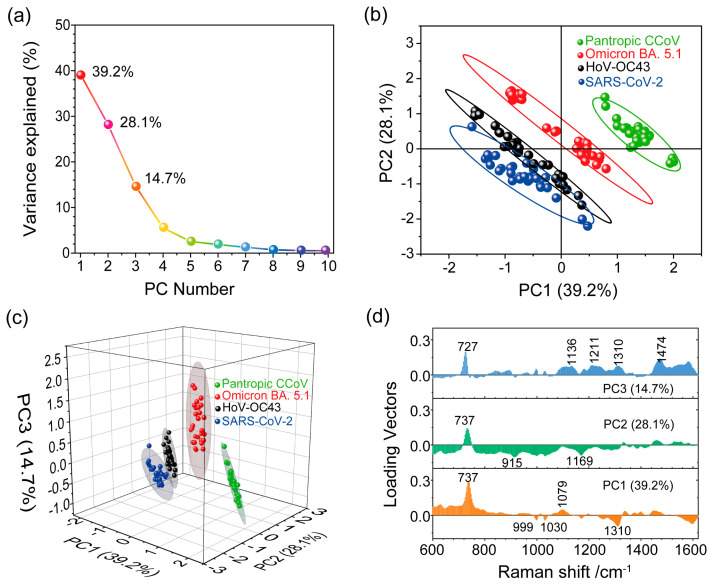
(**a**) Scree plot depicting the percentage of variance explained (*Y*-axis) for the first ten PCs (*x*-axis). (**b**,**c**) Score plot illustrating PCA spectral analysis for the four viruses. (**d**) Loading vector profiles of PC1, PC2 and PC3 for the projected SERS spectra.

To conduct a more systematic analysis of the entire SERS spectral data set obtained from these four types of viruses, we used the PCA method for each data set. The PCA method can not only extract the key spectroscopic information but also capture the differences in the SERS spectra between viral particles. First, we needed to compute the eigenvalues from a covariance matrix to determine the principal components (PCs) of the data. The principal components are given according to the linear combination of variables [54],

(1) $$Y_{i}=a_{i1}X_{1}+a_{i2}X_{2}+\cdots +a_{ip}X_{p}$$

or, in matrix notation,

(2) $$Y_{i}=a_{i}^{T}X$$

where *Y* represents the principal components, *X* represents the variables and *a_i_* represents the weights. As shown in Figure 5a, the explained variance for the ten principal component numbers was obtained based on the normalized SERS spectra of the four types of viruses (see Section S3 in Supplementary Information). We found that the PCA aimed to capture the maximum possible information in the first component, followed by maximum remaining information in the second component and so on. It is important to note that PC1 can explain 39.2% of the total variance, PC2 can explain 28.1% of the total variance and PC3 explains 14.7% of the total variance.

To see whether the first two components contained enough relevant information about the virus samples, we used PC1 and PC2 for spectral data analysis to project the SERS spectra of the different viruses onto distinct spots in the 2D space constructed by PC1 and PC2, as shown in Figure 5b. However, the projected spots of SARS-CoV-2 and HoV-OC43 were not clearly distinguishable, and there was some overlap between them. Ideally, we would choose the number of principal components by adding the explained variance until the sum exceeded 80%. We noticed that the first two components retained less than 70% of the information within the original data. Therefore, we needed to consider the first three principal components (explained over 80% of total variance) to compress spectral data from a multidimensional space into a more manageable 3D subspace, as depicted in Figure 5c. The separation of the projected spots for the four analyzed viruses was evident, indicating significant variations in the surface chemical features among these viruses. This result implies that Raman spectroscopy, in combination with PCA, is a valuable tool for capturing and identifying differences among different pathogens.

After distinguishing the SERS signals of SARS-CoV-2 from the other virus types, we further extracted the essential spectral characteristics unique to each virus. The loading vector profile presented in Figure 5d illustrates the contribution of specific Raman modes crucial for these viruses’ identification. Key contributing Raman modes for PCs exhibit high loading vector values. In the PCA score plot (Figure 5c), the SERS spectra of SARS-CoV-2 are projected in the space where PC1 < 0, PC2 < 0 and PC3 < 0. This indicates that the Raman shifts significantly contributing to negative values of the first three components are important for distinguishing SARS-CoV-2 from the other viruses. By considering these critical Raman shifts leading to negative values of PCs, we identified the Raman shifts at 915 cm^−1^, 999 cm^−1^, 1030 cm^−1^, 1169 cm^−1^ and 1310 cm^−1^ as the most important for SARS-CoV-2 identification. Likewise, specified locations in the score plot (PC1 < 0, PC2 < 0 and PC3 < 0) and (PC1 < 0, PC2 > 0 and PC3 > 0) were occupied by the HoV-OC43 SERS spectra. As depicted in Figure 5d, Raman shifts at 999 cm^−1^, 1030 cm^−1^ and 1310 cm^−1^ (PC1 < 0) significantly contributed to the SERS spectra of HoV-OC43. Furthermore, Raman shifts at 727 cm^−1^ and 737 cm^−1^ played a significant role in identifying, respectively, the Omicron BA. 5.1 variant (PC3 > 0) and the pantropic CCoV (PC1 > 0 and PC2 > 0) among the four viruses. These results imply that the SERS spectroscopy, combined with PCA, enables the detecting and identifying of SARS-CoV-2.

## 4. Conclusions

In this study, we proposed a strategy combining SERS and PCA to detect and identify multiple viruses without the need for target virus-specific probes. A simple, high-performance, reproducible and stable SERS substrate was fabricated by a robust Langmuir–Blodgett self-assembly of Au NPs. Subsequently, SERS spectra of four types of viruses, with a concentration range of 10^2^–10^6^ TCID_50_/mL, were obtained using the Au NPs film substrate. The recorded SERS spectra were rich in spectroscopic information about the viruses, and our method of detection exhibited low LOD values (<100 TCID_50_/mL or even <10 TCID_50_/mL). During the identification process, subtle spectral differences were observed in the SERS spectra of the different viruses. As a result, these small differences in spectra were sufficient to extract key spectral information by using PCA and to distinguish the spectra related to the four types of viruses. The developed virus detection platform based on SERS and PCA has a great potential in detecting COVID-19 and could be extended to use in other public health emergencies and biomedical applications. 

## Figures and Tables

**Table 1:** Summary of SARS-CoV-2 detection methods and LOD.

Target	Methods	LOD	Ref.
SARS-CoV-2 viral RNA	CRISPR/Cas9-Mediated LFA	100 copies per reaction (25 µL)	[37]
SARS-CoV-2	Nanophotonic biosensors	10^2^–10^3^ viruses/mL	[38]
SARS-CoV-2 variant Delta, Omicron, Omicron-BA. 1	CRISPR/Cas12a-empowered SPR platform	15 fM	[39]
SARS-CoV-2 spike protein	Electrochemical	229 fg/mL	[16]
SARS-CoV-2	FET biosensor	~165 copies/mL	[17]
ACE2/RBD	Peptide-modified SERS	300 nM	[40]
SARS-CoV-2 spike protein	MB-labeled AuNPs-Ab-SARS-CoV-2-SERS	0.046 ng/mL	[41]
SARS-CoV-2/influenza A	SERS-LFA strip	5.2 PFU/mL and 23 HAU/mL	[42]
SARS-CoV-2 RNA	DNA-conjugated Ag NR SERS	10^3^ copies/mL	[43]
SARS-CoV-2/H1N1A/Marburg/Zika	Label-free SERS platform	10^3^ copies/mL	[18]
SARS-CoV-2/HoV-OC43/Omicron BA. 5.1/Pantropic CCoV	Label-free SERS platform	23 TCID_50_/mL, 69 TCID_50_/mL, 5 TCID_50_/mL and 17 TCID_50_/mL	This work

**Table 2:** Raman shifts and peak assignments of the four types of viruses.

Raman Shift (cm^−1^)				Peak Assignments	Ref.
SARS-CoV-2	HoV-OC43	Omicron BA. 5.1	Pantropic CCoV		
497	497	497	497	υ (COO-); Phenylalanine, Tyrosine	[44,45]
			630	Tryptophan	[46]
651	651	651		Tyrosine (α-helix)	[44,45]
			661	C-S stretching mode of Cystine	[47]
		727		C-S (protein), CH_2_ rocking, A	[47]
			737	(C-S) stretching	[47,48]
	854	854	854	υ_s_(O-P-O) stretching, T	[44]
	892			DNA/RNA, phosphodiester, deoxyribose, υ(C-COO-)	[44,49]
999	999	999	999	δ(C-C) aromatic ring in Phenylalanine	[44,46]
1030	1030	1030		A, G (DNA/RNA), C=C bending mode of Phenylalanine υs(COO-), Tryptophan	[46,50]
1076	1079			Lipid C-C stretching υ(C-N), υ(PO_2_-) nucleic acid,	[51,52]
			1151	C–N, glycogen	[46]
	1166			C–H in-plane bending band, A, G, C-C/C-N stretching in proteins	[45,53]
1172				δ(C-H), Tyrosine (protein assignment)	[47]
	1211			υ (C-C6H5) of Phenylalanine and Tryptophan	[46]
1310	1310	1310	1310	Amide I, C-H deformation	[44,47]
1446	1446	1446	1446	δ(CH_3_CH_2_), proteins and lipids	[44,45]
1588	1588	1588	1588	A, G, C=C bending mode of phenylalanine, υs(COO-), Tyrosine, Tryptophan	[46,47,48]

## Data Availability

Data is unavailable due to privacy but in case of specific interest by contacting authors will be provided.

## Supplementary Materials

The following supporting information can be downloaded at: https://www.mdpi.com/article/10.3390/bios13121014/s1, Figure S1: SERS spectrum of cell culture medium recorded using the Au NPs film substrate. No significant Raman peaks related to the medium are appreciable; Figure S2: Normalized SERS spectra obtained from four types of viruses (a) SARS-CoV-2, (b) HoV-OC43, (c) SARS-CoV-2 variant Omicron BA. 5.1 and (d) pantropic CCoV for 30 random test dots on four parallel samples. A total of 20 µL of the virus solution (10^4^ TCID_50_/mL) was added to each of substrate and allowed to incubate at room temperature for 2 h and was then used for SERS measurement. Table S1: LOD value conversions across different units of measurement. References [55,56,57,58,59,60,61,62,63,64,65,66] are cited in the supplementary materials.
