# Supplementary material for: The Label-Free Detection and Identification of SARS-CoV-2 Using Surface-Enhanced Raman Spectroscopy and Principal Component Analysis
Source: Biosensors (Basel). 2023 Dec 5;13(12):1014. doi: 10.3390/bios13121014 (PMC10741931; doi:10.3390/bios13121014)
Supplement: Supplementary file 1 [file biosensors-13-01014-s001.zip › biosensors-2663376-supplementary.pdf]

biosensors

MDPI

Supplementary Material

The Label-Free Detection and Identification of SARS-CoV-2 Using Surface-Enhanced Raman Spectroscopy and Principal Component Analysis

Lu Zhou [1,2], Ambra Vestri [1], Valentina Marchesano [1], Massimo Rippa [1], Domenico Sagnelli [1], Gerardo Picazio [3], Giovanna Fusco [3], Jiaguang Han [2], Jun Zhou [4,*] and Lucia Petti [1,*]

S1 Materials and Methods

1. SARS-CoV-2 Omicron Variant BA. 5.1

The sequencing of SARS-CoV-2 Omicron Variant BA. 5.1 viral genome was carried out by Next-Generation Sequencing (NGS) on the MiSeq platform (Illumina) using the MiSeq MicroReagent Kit v2 (300-cycles) (Illumina), according to the previously described method[1]. The consensus sequence was obtained through the software package Geneious R9 (Biomatter) and inserted in the GISAID database (http://www.gisaid.org) available via the accession ID: EPI_ISL_15303201.

The sample was kept in Universal Transport Medium (UTM) (Copan, Brescia, Italy) 3 mL transport medium. Virus isolation and titration was performed in the Class 3 Biosafety Laboratory (BSL3) by infection of the Vero E6 cell line. Vero E6 cells have been kept in EMEM (Gibco, Life Technologies, Europe B.V. Bleiswijk, The Netherlands) supplemented with 10% FBS (Gibco), 1% antibiotic-antifungal (Gibco), 1% l-glutamine (Gibco), grown at 37 ºC and 5% CO_2, and stored in T225 flask. When the cell monolayer has reached 80% confluence, the sample was inoculated using 100 μL of solution, filtered with a 0.45 nm syringe filter (Millipore, Thermo Fisher Scientific, Waltham, MA, USA).

Simultaneously, control cultures were prepared using non-integrated pure EMEM inoculum. After incubation for 1 h to 37 °C, the inoculums were removed and the cells transferred to 25 cm^2 flasks and cultured at 37 °C in EMEM supplemented with 2% FBS, 1% antibiotic-antifungal, 1% l-glutamine. Monolayers were inspected after 24 h under optical microscope for cytotoxic effect observation and, up to 7 days after infection, for cytopathic effect formation (CPE).

Periodically, for a total of 3 weeks, cellular passages were carried out using 100 μL of supernatant as an inoculum for a secondary 25 cm^2 flask. Once the cytopathic effect was verified, the sample was titrated with Endpoint Dilution Assay ($TCID_{50}$/ml) according to the Spearman-Kärber method[2] at a titre of 10^3 $TCID_{50}$/ml, and nucleic acid extraction using MagMAX Viral/Pathogen II Nucleic Acid Isolation Kit, based on magnetic sphere technology. The workflow was automated using kingfisher Flex Magnetic Particle Processor set to 200 μL initial solution and 50 μL final elution, following the manufacturer’s instructions. The eluate was then examined at RT-qPCR for detection and quantification of SARS-CoV-2 which detected a viral load of 4.08 $\times10^8$ copies/μL.

2. Pantropic Canine Coronavirus

Pantropic CCoV isolation was performed from a 2014 sample derived from a pomerianan dog client-owned previously examined by Alfano et. al (2020)[3]. The nucleotide sequence of the analyzed strain was deposited in GenBank under the following accession number: MN086808. The sample was homogenized with phosphate-buffered saline (PBS) and initiated to extraction via MagMAX-like Viral/Pathogen II Nucleic Acid Isolation Kit, following the manufacturer’s instructions. The viral titre was evaluated according to the Spearman-Kärber method[2] and detected a titre of 10^3 $TCID_{50}$/mL. Isolation was performed on A-72 cell line with the same procedure as described above.

The supernatants were recovered and stored in 1 mL aliquots at -80 °C temperature until use. Samples were inactivated at 80 °C for 30 minutes before shipment.

S2 The Calculation of SERS Enhancement Factor for as-Prepared SERS Substrate

The enhancement factor (EF) of the SERS substrate is calculated by using the following equation:[4,5]

$$\mathrm{EF} = (I_{SERS}/I_{bulk}) \times (N_{bulk}/N_{SERS}) \quad \text{(S1)}$$

where I_{SERS} is the integrated intensity of a SERS mode such as the ring-breathing mode (υ(C–S)) at 1079 cm^{-1}, I_{bulk} is the intensity of the same mode in the Raman spectrum of 4MBA, N_{SERS} and N_{bulk} are the numbers of 4MBA molecules on the SERS substrate and the silicon wafer at the laser spot, respectively. The diameter of the laser spot was calculated to be 1.28 μm using the equation[6]: $D_{dimater} = (\lambda/NA) \times 1.22$, in which λ and NA are 785 nm and 0.75 for the specifications of Raman spectrometer, respectively. Besides, the 1.22 factor accounts for the deviation of the laser beam from a perfect Gaussian profile. By our previous works, N_{SERS} was calculated to be 3.9 × 10^{6} based on the following equation[7]: $N_{SERS} = N_A \times A/\sigma$, in which N_A is Avogadro constant, A is effective area occupied by 4MBA molecules under the laser irradiation, and σ=2.0 × 10^{9} cm^{2} mol^{-1} is the per mol area of self-assembled monolayer 4MBA molecules[8]. And N_{bulk} is calculated to be 2.7 × 10^{11} with using 1.5 g·cm^{-3} of 4MBA molecular density and 154.19 g·mol^{-1} of molecular weight. Then, from Figure 1e, I_{bulk} and I_{SERS} are obtained to be 4.2 × 10^{3} and 1.9 × 10^{6}, respectively. As a result, the EF value of the SERS substrate is 3.13 × 10^{7}.

S3. SERS spectra of cell culture medium

SERS spectra of cell culture medium free from viruses (blank) were recorded using a Raman spectrometer (QE Pro-Raman, Ocean Optics) coupled with an Olympus BX51 microscope and a semiconductor laser emitting at a wavelength of 785 nm. An acquisition time of 10 seconds, a 50× objective lens (N.A. = 0.75) and a laser power set within the range of 10 mW were also employed.

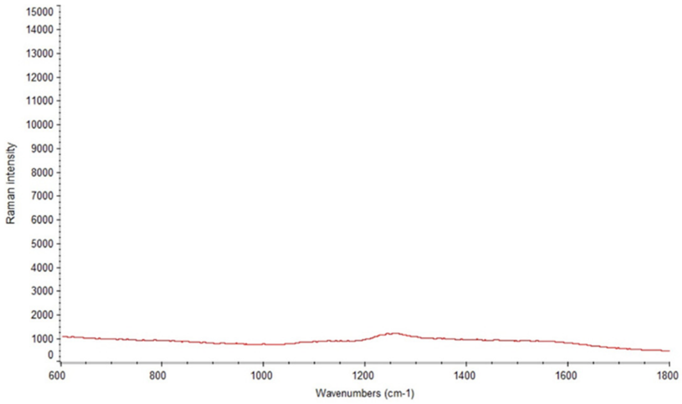

Figure S1 SERS spectrum of cell culture medium recorded using the Au NPs film substrate. No significant Raman peaks related to the medium are appreciable.

S4 CONVERSION TABLE

The LOD values estimated in the manuscript in $TCID_{50}$/mL can be approximately converted into other units of measurement (Table S1) using the following conversions reported in the literature:

1 PFU ≈ 6.69 TCID [9]

1 PFU ≈ 103 viral particles (viruses) [10]

1 virus ≈ 1 fg [10,11]

Table S1 LOD value conversions across different units of measurement.

23 $TCID_{50}$/mL	3.4 PFU/mL	3.4 x 10^3 viruses/mL	3.4 pg/mL	5.6 aM
69 $TCID_{50}$/mL	10.3 PFU/mL	10.3 x 10^3 viruses/mL	10.3 pg/mL	17 aM
5 $TCID_{50}$/mL	0.7 PFU/mL	7 x 10^2 viruses/mL	0.7 pg/mL	1.1 aM
17 $TCID_{50}$/mL	2.5 PFU/mL	2.5 x 10^3 viruses/mL	2.5 pg/mL	4.1 aM

S5 Principal Component Analysis (PCA)

The PCA is a dimensionality reduction method that is often used to reduce the dimensionality of large data set, by transforming a large set of variables into a smaller one that still contains most of the information in the large set[12]. Thus, we employ the PCA method to reduce the dimensionality of the spectroscopic data, which facilitates the identification of variations in the SERS spectra. In our work, PCA was carried out on 120 spectroscopic data sets consisting of 30 spectra on 4 parallel samples obtained from each individual virus (see Figure S2). In general, before performing PCA, the spectral data should be normalized. The reason is that the spectra information mainly comes from the relative intensity and Raman shift, and the absolute intensity of the peak is related to the laser power fluctuation and focusing, the SERS substrate differences and the fluorescence background, the normalization can eliminate these effects. On the other hand, PCA model is quite sensitive regarding the variances of the initial data. That is, if there are large differences between the initial data intensity, those variables with larger signal intensity will dominate over those with smaller signal intensity which will lead to biased results[13]. So, the first step in PCA is to normalize each spectra data, as shown in Figure S2. The SERS spectral region is between 600 and 1620 cm^{-1} was used to perform PCA to identify the maximum variations of each data set. This is because that there is a strong SERS signal in 525 cm^{-1}, origins from the Silicon wafer, which will have a great impact on the subsequent PCA training. And there is no signature SERS signal when Raman shift is greater than 1620 cm^{-1}, which will only increase the numbers of variables in performing PCA. The results of PCA are given in the main text.

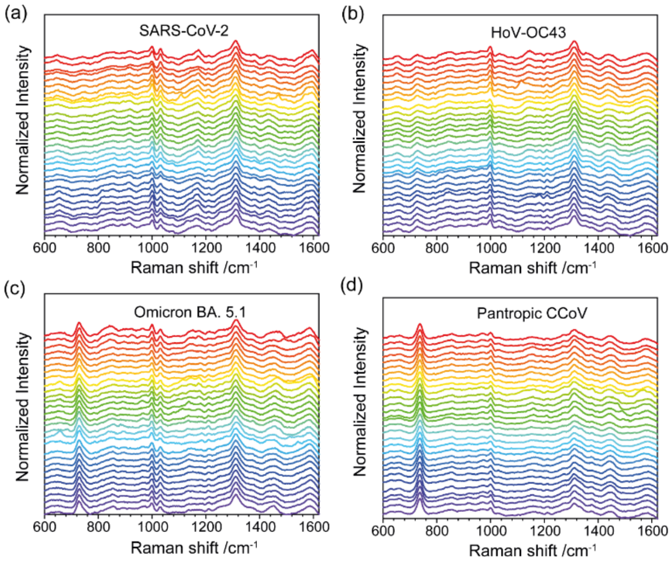

Figure S2 Normalized SERS spectra obtained from four types of viruses (a) SARS-CoV-2, (b) HoV-OC43, (c) SARS-CoV-2 variant Omicron BA. 5.1 and (d) Pantropic CCoV for 30 random test dots on

4 parallel samples. 20 μL of the virus solution (10^4 $TCID_{50}$/mL) was added to each of substrate and allowed to incubate at room temperature for 2 h, and then for SERS measurement.

References

(1) Cicchitto, G.; Cardillo, L.; De Martinis, C.; Sabatini, P.; Marchitiello, R.; Abate, G.; Rovetti, A.; Cavallera, A.; Apuzzo, C.; Ferrigno, F.; Fusco, G. Effects of Casirivimab/Imdevimab Monoclonal Antibody Treatment among Vaccinated Patients Infected by SARS-CoV-2 Delta Variant. *Viruses* **2022**, *14* (3), 650.

(2) Kärber, G. Beitrag zur kollektiven Behandlung pharmakologischer Reihenversuche. *Naunyn-Schmiedebergs Arch. Für Exp. Pathol. Pharmakol.* **1931**, *162* (4), 480-483.

(3) Alfano, F.; Fusco, G.; Mari, V.; Occhiogrosso, L.; Miletti, G.; Brunetti, R.; Galiero, G.; Desario, C. C.; Cirilli, M.; Decaro, N. Circulation of Pantropic Canine Coronavirus in Autochthonous and Imported Dogs, Italy. *Transbound. Emerg. Dis.* **2020**, tbed.13542.

(4) Wang, Y.; Sun, Z.; Hu, H.; Jing, S.; Zhao, B.; Xu, W.; Zhao, C.; Lombardi, J. R. Raman Scattering Study of Molecules Adsorbed on ZnS Nanocrystals. *J. Raman Spectrosc.* **2007**, *38* (1), 34-38.

(5) Camargo, P. H. C.; Au, L.; Rycenga, M.; Li, W.; Xia, Y. Measuring the SERS Enhancement Factors of Dimers with Different Structures Constructed from Silver Nanocubes. *Chem. Phys. Lett.* **2010**, *484* (4–6), 304-308.

(6) He, Y.; Su, S.; Xu, T.; Zhong, Y.; Zapien, J. A.; Li, J.; Fan, C.; Lee, S.-T. Silicon Nanowires-Based Highly-Efficient SERS-Active Platform for Ultrasensitive DNA Detection. *Nano Today* **2011**, *6* (2), 122-130.

(7) Jia, H.; Zeng, J.; Song, W.; An, J.; Zhao, B. Preparation of Silver Nanoparticles by Photo-Reduction for Surface-Enhanced Raman Scattering. *Thin Solid Films* **2006**, *496* (2), 281-287.

(8) Wang, A.-J.; Lv, J.-J.; Zhou, D.-L.; Weng, X.; Qin, S.-F.; Feng, J.-J. Facile Synthesis of Ultrathin Worm-like Au Nanowires for Highly Active SERS Substrates. *New J Chem* **2014**, *38* (8), 3395-3400.

(9) L. Shan, D. Yang, D. Wang, and P. Tian, "Comparison of cell-based and PCR-based assays as methods for measuring infectivity of Tulane virus," *Journal of Virological Methods*, vol. 231, pp. 1–7, 2016, doi: 10.1016/j.jviromet.2016.01.012.

(10) R. Sender *et al.*, "The total number and mass of SARS-CoV-2 virions," *Proceedings of the National Academy of Sciences of the United States of America*, vol. 118, no. 25, pp. 1–9, 2021, doi: 10.1073/pnas.2024815118.

(11) J. H. Choe, "Two weeks," *Annals of Internal Medicine*, vol. 172, no. 10, pp. 697–698, 2020, doi: 10.7326/M20-1190.

(12) Jolliffe, I. T.; Cadima, J. Principal Component Analysis: A Review and Recent Developments. *Philos. Trans. R. Soc. Math. Phys. Eng. Sci.* **2016**, *374* (2065), 20150202.

(13) Ullah, R.; Khan, S.; Chaudhary, I. I.; Shahzad, S.; Ali, H.; Bilal, M. Cost Effective and Efficient Screening of Tuberculosis Disease with Raman Spectroscopy and Machine Learning Algorithms. *Photodiagnosis Photodyn. Ther.* **2020**, *32*, 101963.
